# Straw returning enhances oxygen radical scavenging capacity by modulating of AsA–GSH pathway in maize subjected to water deficit

**DOI:** 10.3389/fpls.2026.1857578

**Published:** 2026-08-25

**Authors:** Yan Li, Jiying Sun, Jian Liu, Zhiqiang Yuan, Yafang Fan, Chen Sun, Shaodong Wang, Yanli Zhang

**Affiliations:** 1 College of Agronomy, Inner Mongolia Agricultural University, Hohhot, China; 2 Institute of Crop Science, Inner Mongolia Academy of Agricultural & Animal Husbandry Sciences, Hohhot, China; 3 Vocational and Technical College, Inner Mongolia Agricultural University, Baotou, China; 4 Science and Technology Park, Inner Mongolia Agricultural University, Hohhot, China

**Keywords:** antioxidant defense system, drought tolerance, maize, straw returning, yield

## Abstract

**Introduction:**

Global warming is altering crop growing conditions across diverse climate zones worldwide. In particular, drought stress, a major abiotic constraint, severely restricts the potential for maize yield improvement. The implementation of effective soil improvement and water–saving agronomic practices has emerged as a key measure for mitigating climate change and promoting sustainable agricultural development. This study aims to systematically investigate the effects of straw returning on maize grain yield under water–limited conditions by improving soil properties and modulating the ascorbate–glutathione (AsA–GSH) cycle.

**Methods:**

This study was conducted over a two–year field period using six maize varieties–DK159, MC703, A2636, 303×178, ZQ1, and ZJ330–as experimental materials. The experiment employed tillage methods as the primary treatment, with two levels: straw returning (ST) and traditional plowing (TP, without straw return). Based on this, two water management regimes were implemented: drought stress (DS) and normal irrigation (NI). This study analyzes the differential responses of various maize varieties under the interaction of different tillage practices and moisture conditions.

**Results:**

Straw returning can effectively improve soil conditions under drought stress, by regulating the levels of antioxidants and the activities of antioxidant enzymes in the AsA–GSH cycle, it promotes the efficient scavenging of reactive oxygen species (ROS), reduces oxidative damage to crops caused by environmental stress, and thereby helps maintain relatively stable yields. This study indicates that, compared with traditional plowing, straw returning increased maize yields by 0.42%–29.64% under drought stress. Among the various treatment combinations, straw returning (ST) + normal irrigation (NI) Treatment Combination produced the best soil quality and the highest yield.

**Implications:**

The findings of this study provide theoretical support for improving farmland soil quality, enhancing maize drought tolerance, and maintaining grain yield, while also laying a foundation for further research on crop responses to drought stress.

## 1 Introduction

As one of the world’s three major staple crops, maize is not only a key source of human food but also an essential raw material for poultry and livestock feed, food processing, industrial applications, and bioenergy production. It plays an irreplaceable role in ensuring national food security and supporting economic development (Zhou et al., 2024). In recent years, driven by the development of high–yielding varieties, improvements in cultivation practices, and advances in agricultural technology, both the per–unit–area yield and total production of maize in China have continued to increase steadily (Zhao and Wang, 2013). In 2024, China’s maize planting area reached 44.9333 million ha, with a total output of 299 million metric tons, accounting for 28.5% of global production and reinforcing China’s position as the world’s second–largest maize producer. In particular, the total maize planting area in Inner Mongolia reached 4.4 million ha, with an average yield of 7,627.5 kg per ha, ranking fifth nationwide, this region is one of China’s major maize–producing areas (Inner Mongolia: Agricultural Technology Promotion Breaks Ground, 2025). As maize yields increase, the amount of maize straw produced also rises accordingly. In 2024, maize straw production in the region reached 37.65 million metric tons, with a recoverable amount of 34.71 million metric tons. The rational utilization of crop straw resources is essential for the sustainable development of agriculture, as improper management can result in severe air pollution and substantial resource waste (Krishna and Mkondiwa, 2023).

As an important soil improvement practice, straw returning not only recycles the nutrients contained in crop residues back to the farmland, thereby effectively maintaining the material cycling of agroecosystems, but also improves soil physical properties and enhances overall soil quality (Zhang et al., 2025). Studies have shown that straw returning can significantly improve soil aggregate structure by promoting the formation and stability of macro–aggregates, thereby reducing soil bulk density and increasing soil porosity, aeration, and permeability. Consequently, it enhances the soil’s water and nutrient–holding capacity as well as its buffering capacity, creating a more favorable soil environment for crop growth (Chen et al., 2022). In most regions of China, straw returning is widely recognized as an important practice for sustainable agricultural development. Owing to its significant contributions to soil moisture conservation and water retention, as well as to stabilizing crop yields (Yin et al., 2022; Yu et al., 2022; Wang et al., 2025a), this practice has attracted increasing attention in the field of agricultural water resource management. Other sustainable soil management practices, including biochar and organic amendments, have likewise been shown to improve maize growth, physiological performance, and productivity under water–deficit conditions, further emphasizing the importance of integrated soil management strategies for enhancing drought resilience (Fatima et al., 2024).

The climate crisis driven by global warming has increased the frequency, duration, and intensity of drought stress, making drought an increasingly severe challenge in the arid and semi–arid regions of Inner Mongolia and severely constraining the sustained and stable growth of crop yields (Ali et al., 2024; Ameen et al., 2024). In addition, drought stress can severely disrupt plant water balance and metabolic homeostasis (Ding et al., 2023). To maintain normal physiological functions, plants must coordinate the production and scavenging of ROS to keep their levels within a normal range in cells and organelles (Mansoor et al., 2022). The AsA–GSH cycle directly reduces and detoxifies excess hydrogen peroxide (H_2_O_2_) in plants. The two non–enzymatic antioxidants, AsA and GSH, generated through this metabolic pathway, can also scavenge various ROS, such as superoxide anions (O_2_^-^) and singlet oxygen (¹O_2_). Through a multi–pathway synergistic ROS scavenging mechanism, this cycle alleviates oxidative damage induced by drought stress, thereby effectively preserving the structural integrity of cell membranes and maintaining normal physiological functions (Ma et al., 2026).

To address challenges such as water scarcity during the maize growing season under climate change, it is increasingly important to develop management strategies that can simultaneously improve soil physical properties, enhance resource use efficiency, and stabilize crop yields. Straw returning as a typical green agricultural practice, can improve soil porosity and enhance soil water and nutrient retention capacity, thereby serving as an effective strategy for alleviating crop drought stress. However, most existing studies have focused on the effects of single practices, such as straw returning or irrigation, on soil water and nutrient dynamics, crop growth, and yield (Bandurska, 2022; Nasir and Toth, 2022). The synergistic effects of straw returning combined with irrigation on improving soil structure, enhancing crop drought resistance, and increasing productivity remain unclear, and the underlying physiological mechanisms require further investigation. Based on this, this study evaluates indicators including comprehensive soil quality, non–enzymatic antioxidants in the AsA–GSH cycle, key antioxidant enzyme activities, and grain yield. This study reveals how straw returning alleviates oxidative damage in maize under drought stress, clarifies the positive role of the AsA–GSH cycle in scavenging ROS under straw returning regulation, and elucidates its effects on grain yield and yield components. This study provides a theoretical basis and scientific support for achieving high and stable maize yields in China’s major grain–producing areas, promoting the efficient utilization of agricultural by–products in the arid and semi–arid regions of northern China, and advancing the green and sustainable development of farmland systems.

## 2 Materials and methods

### 2.1 Experimental site overview

**Figure 1:**
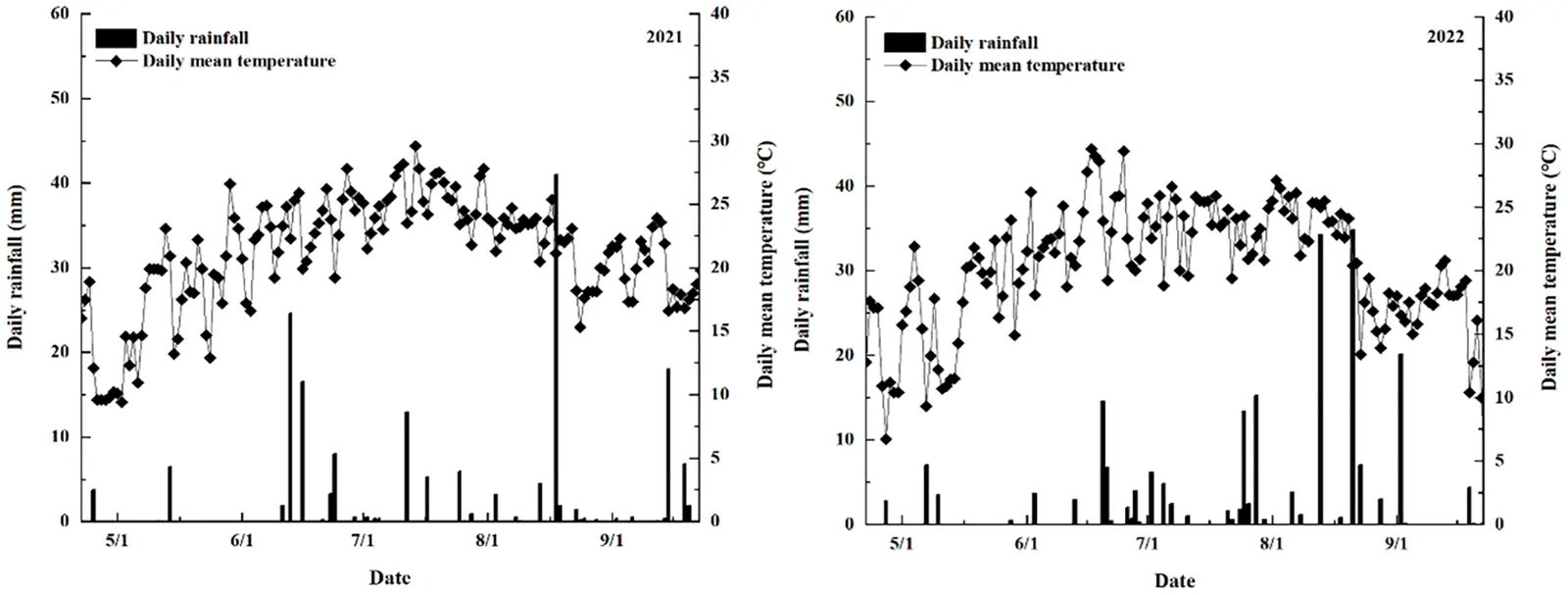
Daily average temperature and rainfall at the site.

The study was conducted at the Maize Center of Inner Mongolia Agricultural University, located within the Vocational and Technical College of the university (40°33′ N, 110°31′ E), Tumote Right Banner, Baotou City, at an altitude of 1008.62 m, during 2021–2022. The site lies in the Tumochuan Plain Irrigation District and has a temperate continental monsoon climate, with a frost–free period of 150 days and an annual average temperature of 6–8 °C. The soil was sandy loam, and its basic fertility is summarized in **Table 1**. Daily temperature and precipitation during the experiment are shown in **Figure 1**. In 2021, the reproductive period received 169.60 mm of rainfall, mainly from June to August, with an average temperature of 20.74 °C. In 2022, rainfall during the reproductive period totaled 205.80 mm, primarily in August and September, with an average temperature of 20.45 °C.

**Table 1:** Characteristics of the experimental site.

Year	Organic matter (g/kg)	Alkaline N (mg/kg)	Alkaline P (mg/kg)	Exchangeable K (mg/kg)	pH
2021	12.06	29.33	12.80	113.75	8.12
2022	13.78	24.39	13.30	121.85	8.20

### 2.2 Experimental materials and design

The experiment used a split–split plot design. Straw returning (factor A) was set as the main plot, irrigation treatments (factor B) as the subplot, and maize varieties (factor C) as the sub–subplot.

The main plot (factor A) included two treatments: A1 represents straw returning (ST), in which maize residue from the 2021–2022 season was chopped into 3–5 cm pieces using a horizontal crushing and returning machine after harvest, evenly spread, and incorporated into the soil at 35 cm depth with a deep–turning machine at 12 t/hm²; A2 represents traditional plowing (TP), where all maize residue was removed from the field after harvest.

The subplot (factor B) included two irrigation levels based on field capacity (FC): B1 represents drought stress (DS, 45% FC), with water stress imposed for a total of four weeks, including two weeks before and two weeks after the silking stage(During the silking period, FC was maintained by adjusting irrigation using a moisture flow meter (SZLDE–L; Shanghai, China), while a multipoint soil temperature and humidity recorder (JL–01; QYE, Handan, China) and a soil moisture tester (WKT–M101; Viscometer, Jiangsu, China) monitored soil moisture in real time to achieve the target water gradient.); B2 represents normal irrigation (NI, 75% FC), with standard water management consisting of four irrigations during the growing season: at the jointing stage, the large bell stage, the silking stage, and 10 days after silking. Each irrigation was applied at a rate of 750 m³/hm.

The sub–subplot (factor C) included six maize varieties: MC703, A2636, DK159, ZQ1, 303×178, and ZJ330 (These varieties were selected based on drought tolerance screening trials and represent different levels of drought resistance. Low drought–tolerant varieties included MC703 and ZQ1, moderately drought–tolerant varieties included A2636 and 303×178, and highly drought–tolerant varieties included DK159 and ZJ330).

The 24 treatment combinations in this study are presented in **Table 2**. Each treatment was replicated three times, resulting in a total of 72 plots. Each experimental plot measured 6 m×5 m (length× width).

**Table 2:** Experimental factors and treatments.

Order number	Factor A	Factor B	Factor C	Treatment combination
1	Straw returning (ST)	Drought stress (DS: 45%FC)	MC703	A_1_B_1_C_1_
2			A2636	A_1_B_1_C_2_
3			DK 159	A_1_B_1_C_3_
4			ZQ 1	A_1_B_1_C_4_
5			303×178	A_1_B_1_C_5_
6			ZJ330	A_1_B_1_C_6_
7		Normal irrigation (NI: 75%FC)	MC703	A_1_B_2_C_1_
8			A2636	A_1_B_2_C_2_
9			DK 159	A_1_B_2_C_3_
10			ZQ 1	A_1_B_2_C_4_
11			303×178	A_1_B_2_C_5_
12			ZJ330	A_1_B_2_C_6_
13	Traditional plowing (TP)	Drought stress (DS: 45%FC)	MC703	A_2_B_1_C_1_
14			A2636	A_2_B_1_C_2_
15			DK 159	A_2_B_1_C_3_
16			ZQ 1	A_2_B_1_C_4_
17			303×178	A_2_B_1_C_5_
18			ZJ330	A_2_B_1_C_6_
19		Normal irrigation (NI: 75%FC)	MC703	A_2_B_2_C_1_
20			A2636	A_2_B_2_C_2_
21			DK 159	A_2_B_2_C_3_
22			ZQ 1	A_2_B_2_C_4_
23			303×178	A_2_B_2_C_5_
24			ZJ330	A_2_B_2_C_6_

In this experiment, maize was planted at a density of 82,500 plants/ha, with a plant spacing of 0.2 m and a row spacing of 0.6 m. Basal fertilization included ammonium dihydrogen phosphate (N 18%, P_2_O_5_ 46%) at 375 kg/ha and potassium sulfate (K_2_O 51%) at 150 kg/ha applied before seeding. Urea (N 46%) was applied as a supplementary fertilizer: 30% at V6, 60% at V12, and 10% at R2, totaling 345 kg/ha. The total annual nutrient application per hectare was 226.2 kg N, 76.5 kg K_2_O, and 172.5 kg P_2_O_5_. Weed and pest management followed local field practices throughout the maize reproductive period.

### 2.3 Measurement indicators and methods

#### 2.3.1 Soil nutrient index

On the sowing date, soil samples from 0–40 cm depth were collected from each treatment. Samples were air–dried in a cool place, crushed, and sieved through 0.15–0.25 mm mesh. Different particle–size fractions were then used for the determination of essential soil nutrients according to standard protocols (Nguyen et al., 2020).

The parameters and procedures are listed below.

1. Organic matter was determined using the potassium dichromate titration method.
2. Available N was determined using the alkaline hydrolysis diffusion–absorption method.
3. Olsen P was determined using the NaHCO_3_ (0.5 mol/L) Mo–Sb colorimetric method.
4. Exchangeable K was determined using the flame photometric method.
5. The soil pH value was determined using the pH potentiometer method (using H_2_O as the extractant).

#### 2.3.2 Comprehensive evaluation parameters for soil physical properties

(1) Soil moisture content: During the silking stage, soil samples were collected using aluminum boxes from the 0–20 cm soil layers, with three replicates taken for each depth. After sampling, the soils were oven–dried to determine soil moisture content (Hossain et al., 2010). Soil moisture content was calculated using the following formula:


$$Y=\frac{\left(m_{1}-m_{2}\right)}{\left(m_{2}-m_{0}\right)}\times 100$$

In this equation, Y represents soil moisture content (%); m_0_ represents the weight of the aluminum box (g); m_1_ represents the weight of the aluminum box plus wet soil (g); and m_2_ represents the weight of the aluminum box plus dry soil (g).

(2) Soil bulk density: During the silking stage, undisturbed soil samples were collected from the 0–20 cm soil layer using a 100 cm³ ring knife. Three replicates were taken along a horizontal section of the field profile. After sampling, the soils were transported to the laboratory, carefully cleaned, and weighed while fresh. The samples were then oven–dried at 105–110 °C for 16 h. After drying, they were cooled and weighed to obtain their dry mass (Bao, 2000). Finally, the soil was removed and the ring knife was weighed.


$$pb=\frac{\left(M-G\right)}{V}$$

In this equation, ρb represents soil bulk density (g/cm³); M is the combined weight of the ring cutter and dry soil (g); G is the weight of the ring cutter (g); and V is the volume of the ring cutter (100 cm³).


Soil porosity=(1−pb/2.65)×100 

The comprehensive soil quality index was calculated based on the measurement results in (1) and (2) (Bao, 2000).

(3) Soil three comparisons: Solid phase = 100 – ; Liquid phase = soil moisture content; Gas phase = porosity – soil moisture content. The ideal ratio for farmland soil is solid phase: liquid phase: gas phase = 50:25:25.

(4) General soil structure index (GSSI):


$$GSSI={\left[X-25\times Y\times Z\right]}^{0.4769}$$

(5) Soil triple–phase structure distance (STPSD):


$$STPSD=\sqrt{X-50^{2}+X-50Y-25+Y-25^{2}}$$

In the equation, X represents the soil solid phase value; Y represents the soil liquid phase value; Z represents the soil gas phase value.

#### 2.3.3 H_2_O_2_ and O_2_^–^ contents

H_2_O_2_ was determined following Ruch (Ruch et al., 1989). Briefly, the extract was mixed with potassium phosphate buffer and potassium iodide, and absorbance was measured at 230 nm. O_2_^-^ was determined according to Zhang (Zhang et al., 2022) by reacting the supernatant with phosphate buffer, hydroxylamine hydrochloride, p-amino benzene sulfonic acid, and α-naphthylamine, followed by ether extraction, and measuring absorbance at 530 nm.

#### 2.3.4 AsA–GSH cycle antioxidant content

(1) Ascorbic acid (AsA): Measurements are performed using the colorimetric method using three maize leaf samples (Turcsanyi et al., 2000). Samples (0.5 g) were homogenized in 20 mL of 50 g/L trichloroacetic acid (TCA) on ice and brought to 100 mL with TCA. After 10 min extraction, the mixture was filtered and the filtrate collected. For determination, 1 mL of extract was combined with 1 mL TCA (50 g/L), 1 mL ethanol, 0.5 mL 0.4% phosphoric acid–ethanol, 1 mL 5 g/L BP–ethanol, and 0.5 mL 0.3 g/LFeCl_3_–ethanol. The mixture was incubated at 30 °C for 60 min, and absorbance was recorded at 534 nm. AsA content was calculated from a standard curve.

(2) Dehydroascorbic acid (DHA): Measurements are performed using the colorimetric method (Takahama and Oniki, 1992). Briefly, 1 mL of the sample extract (prepared for AsA determination) was mixed with 0.5 mL of 60 mmol/L DTT–acetic acid solution and adjusted to pH 7–8 with NaHPO_4_–NaOH. The mixture was incubated at room temperature for 10 min to reduce DHA. Afterward, 0.5 mL of 0.2 g/mL trichloroacetic acid was added, and the pH was adjusted to 1–2. Total ascorbic acid was then measured as for AsA, and DHA content was calculated by subtracting AsA from the total AsA.

(3) Reduced glutathione (GSH): Determined by spectrophotometry (Hissin and Hilf, 1973). 0.1 g of fresh sample was ground on ice in a pre–chilled mortar with 750 μL of extraction medium (5% w/v TCA, 1 mmol/L EDTA) and a small amount of quartz sand. The homogenate was transferred to a 5 mL centrifuge tube, and the mortar was rinsed with an additional 750 μL of extraction medium, which was combined with the homogenate. The mixture was centrifuged at 15,000 rpm for 15 min at 4 °C, and the supernatant was used as the GSH test solution. Absorbance at 412 nm was measured using a spectrophotometer with the following setup: Cell 1–distilled water (zero adjustment); Cell 2–blank control from the standard curve; Cell 3–1 mL sample + 1 mL PBS + 0.5 mL DTNB, mixed thoroughly before measurement.

(4) Oxidized glutathione (GSSG): Determined by spectrophotometry (Diao et al., 2023). Briefly, 0.5 g of plant tissue was homogenized in 5 mL of pre–chilled phosphate buffer. The homogenate was centrifuged at 10,000 rpm for 15 min at 4 °C, and the supernatant was collected. A 1 mL aliquot of supernatant was mixed with 50 μL of 0.2 mol/L NEM and incubated at room temperature for 1 h. Then, 0.5 mL of the NEM–treated extract was combined with 2.5 mL phosphate buffer and 0.5 mL DTNB solution. Absorbance was measured at 412 nm to calculate GSSG content.

#### 2.3.5 AsA–GSH cycle enzyme activity

All enzymatic assays were conducted using the same crude extract. The extraction procedures were consistently performed at 4 °C to ensure sample stability and prevent enzyme degradation. The samples were ground in liquid nitrogen and homogenized for 10 minutes in 50 mM sodium phosphate buffer (pH 7.0) supplemented with 0.2 mM ethylenediaminetetraacetic acid (EDTA) and 20% polyvinylpolypyrrolidone (PVPP). The resulting homogenates were filtered and centrifuged at 15000 rmp for 20 minutes at 4 °C. The supernatant was subsequently desalted using a Sephadex G–50 column, following the method described by (Helmerhorst and Stokes, 1980).

1. Ascorbic acid oxidase (AAO) activity was assayed following the method described by Cardello (Cardello et al., 1998). For the assay, 160 µL of extract was incubated with 400 µL of 0.1 M sodium phosphate buffer (pH 5.5) and 400 µL of 1.0 mM AsA solution at room temperature. The reaction was stopped by adding 2.4 mL of 0.2 N hydrochloric acid every 30 s for 3 min, totaling seven readings. Absorbance was measured at 244 nm using a spectrophotometer.
2. Ascorbate peroxidase (APX) activity was determined by monitoring the decrease in absorbance at 290 nm, resulting from the oxidation of AsA over a period of 5–10 minutes, as described by Nakano and Asada (1981) (Nakano and Asada, 1981). The reaction mixture consisted of 1 mL of 0.68 mmol/L AsA and 0.1 mmol/L EDTA in 0.1 mol/L sodium phosphate buffer (pH 7.0), 1 mL of 4 mmol/L H_2_O_2_, and 50–100 μL enzyme extract.
3. Monodehydroascorbate reductase (MDHAR) activity was assayed following the method described by Zhang and Kirkham (Zhang and Kirkham, 1996). The reaction mixture comprised 50 mmol/L sodium phosphate buffer (pH 7.6), 2.5 mmol/L AsA, 4 units of AsA oxidase, 50 μL of enzyme extract, and 0.1 mmol/L NADH. The oxidation of NADH was monitored by measuring the absorbance at 340 nm.
4. Dehydroascorbate reductase (DHAR) activity was determined using the method described by Arrigoni (Arrigoni et al., 1992). The assay mixture consisted of 0.9 mL of 0.05 mol/L/potassium phosphate buffer (pH 6.3), 100 mL of 13.5 mmol/L GSH, 100 mL of 13.5 mmol/L DHA, and 100–200 mL of enzyme extract. The enzymatic activity was quantified by measuring the production of ascorbate at a wavelength of 265 nm over a 5–minute period.
5. Glutathione reductase (GR) activity was measured using the method outlined by Grill (Hermann and Dieter, 1978), which involved monitoring the oxidation rate of NADH at a wavelength of 340 nm. The reaction mixture comprised 0.5 mmol/L NADPH, 10 mmol/L GSSG, 10 mmol/L EDTA dissolved in 0.1 mol/L/sodium phosphate buffer (pH 7.8), and 50–100 mL of enzyme extract.

#### 2.3.6 Yield estimation and variety evaluation

Prior to harvest, grain yield measurements were conducted for each treatment. Border rows were excluded to minimize edge effects, and two central rows from each plot were selected for sampling. A 5 m section within each row was harvested, and ten ears were randomly collected for the assessment of yield components. The measured parameters included ear length, ear diameter, barren tip length, number of rows per ear, number of grains per row, and hundred–kernel weight. For the determination of actual grain yield, all ears harvested from each plot were threshed and weighed. Grain moisture content was determined with a PM8188 grain moisture meter, and yield was standardized to 14% moisture (Yang et al., 2022).


$$Grain\;yield\;(kg\cdot ha^{-1})=maize\;grain\;weight\;(kg\cdot m^{-2})\times 10000\;(m^{2})$$

### 2.4 Statistical analysis

Data were processed using Microsoft Excel 2010 (Microsoft, Redmond, WA, USA). A three–way analysis of variance (ANOVA) was performed using IBM SPSS Statistics 25.0 (IBM, New York, NY, USA). Results are presented as mean ± standard deviation, and differences between means were evaluated by Tukey’s HSD post–hoc test at a significance level of 0.05.

## 3 Result

### 3.1 Straw returning improves soil environment under drought stress

#### 3.1.1 Analysis of variance of soil physical properties

As shown in **Table 3**, the results of the analysis of variance (ANOVA) for the comprehensive soil physical evaluation index across the two experimental years indicate that: In 2021 and 2022, the three main factors–tillage method, irrigation treatment, and variety–each exerted highly significant effects (P < 0.001) on both the soil triple–phase structure distance (STPSD) and the general soil structure index (GSSI). From the perspective of two–factor interactions, several interaction terms reached statistical significance in different years or for different evaluation indicators, with their patterns of significance varying to some extent across conditions. The three–way interaction among the factors reached statistical significance only in certain years and for specific soil structure indicators, notably exerting a significant effect on STPSD in 2021 and a highly significant effect on GSSI in 2022. This suggests that the significance of the interaction varies depending on both the year and the evaluation indicator.

**Table 3:** ANOVA results for soil physical properties across different treatments (F–value).

Year	Source of variation	STPSD	GSSI
2021	Tillage method(T)	292.98**	246.43**
	Irrigation treatment(I)	1255.90**	827.64**
	Variety(V)	504.91**	334.62**
	T×I	4.33*	0.43ns
	T×V	1.55ns	0.83ns
	I×V	44.16**	4.42*
	T×I×V	3.75*	0.96ns
2022	Tillage method(T)	175.82**	515.34**
	Irrigation treatment(I)	795.37**	2499.19**
	Variety(V)	273.52**	809.51**
	T×I	38.17**	54.59**
	T×V	2.60*	9.77**
	I×V	4.83**	13.98**
	T×I×V	1.08ns	7.87**

(A) soil triple–phase structure distance (STPSD). (B) general soil structure index (GSSI). Each parameter was measured in triplicate. Statistical differences among groups were assessed using one-way ANOVA followed by Tukey’s multiple-comparisons test. All pairwise comparisons were performed. ns = not significant (p > 0.05), *significant (p < 0.05), **highly significant (p < 0.001). T, tillage method; ST, straw returning; TP, traditional plowing without straw; I, irrigation treatment; NI, normal irrigation, 75% FC; DS, drought stress, 45% FC; V, variety (MC703, A2636, DK159, ZQ1, 303×178, ZJ330).

#### 3.1.2 Straw returning improves soil triple–phase composition, STPSD, and GSSI under drought stress

**Figure 2:**
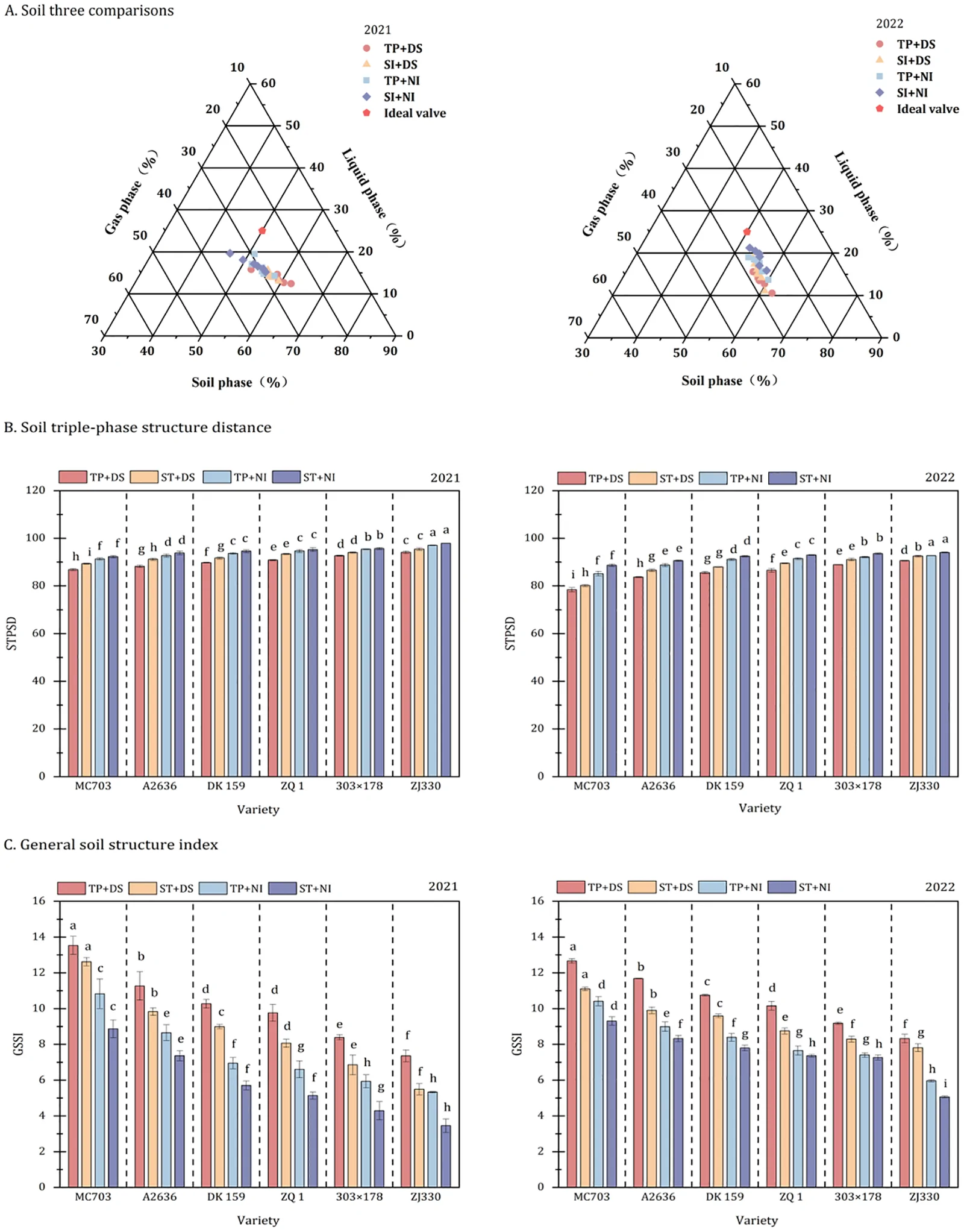
Responses of soil physical properties to straw returning and irrigation amount. **(A)** soil triple–phase composition (solid phase, liquid phase, and gas phase). **(B)** soil triple–phase structure distance (STPSD). **(C)** general soil structure index (GSSI). For a given treatment, different lowercase letters indicate significant differences among treatments (p < 0.05). Error bars represent standard deviation (SD) based on three replicates. Statistical differences among groups were assessed using one-way ANOVA followed by Tukey’s multiple-comparisons test. All pairwise comparisons were performed. The subplots within each figure were analyzed independently. TP, traditional plowing (without straw); ST, straw returning; NI, normal irrigation (75% FC); DS, drought stress (45% FC).

**Figure 2** presents the combined effects of straw returning and irrigation rates on soil three–phase composition, STPSD, and GSSI over the two year experimental period. The results indicate that under low irrigation rates (45% FC), the deviation of the soil phase ratio from the optimal condition for cultivated soil (50% solid, 25% liquid, and 25% gas phase) increases; However, under straw returning (ST) treatment, the proportions of the solid, liquid, and gas phases more closely approximated the ideal soil composition. Under straw returning (ST) treatment, compared with normal irrigation (75% FC), drought stress (45% FC) increased STPSD by 2.34%–8.61% and decreased GSSI by 17.75%–32.38%; Under traditional plowing (TP) treatment, compared with normal irrigation (75% FC), drought stress increased STPSD by 1.67%–10.63% and decreased GSSI by 12.43%–37.45%. It is worth noting that, compared with traditional plowing (TP), the variations in STPSD and GSSI were smaller under straw returning (ST) treatment; among the varieties, the response was most pronounced in DK159, whereas ZJ330 showed the weakest response.

### 3.2 Straw returning enhances ROS scavenging capacity under drought stress

#### 3.2.1 Analysis of variance of H_2_O_2_ and O_2_^-^

As shown in **Table 4**, the results of the ANOVA for ROS levels in maize leaves across the two experimental years indicate that the three main factors–tillage method, irrigation treatment, and variety–all had highly significant effects (P < 0.001) on the concentrations of H_2_O_2_ and O_2_^-^ in maize leaves. Regarding the two–factor interactions, those between tillage × irrigation, tillage × variety, and irrigation × variety were statistically significant for certain years and ROS indicators, with significance varying across years and indicators. The three–factor interaction was statistically significant only in specific years and for certain ROS indicators. It showed a significant effect on H_2_O_2_ accumulation in 2021, a highly significant effect on H_2_O_2_ accumulation in 2022, and a highly significant effect on O_2_^-^ accumulation in both experimental years. These results indicate that different ROS indicators respond differently to the three–factor interaction.

**Table 4:** ANOVA results for reactive oxygen species across different treatments (F–value).

Year	Source of variation	H_2_O_2_ (umol/g)	O_2_^–^ (umol/ml)
2021	Tillage method(T)	915.57**	1715.16**
	Irrigation treatment(I)	4501.85**	6525.57**
	Variety(V)	391.40**	653.53**
	T×I	6.95*	107.20**
	T×V	3.45*	37.73**
	I×V	15.98**	75.60**
	T×I×V	3.97*	58.79**
2022	Tillage method(T)	115.07**	454.90**
	Irrigation treatment(I)	919.81**	1411.68**
	Variety(V)	164.37**	337.57**
	T×I	1.65ns	98.90**
	T×V	4.10*	46.60**
	I×V	12.99**	90.63**
	T×I×V	5.13**	70.60**

(A) hydrogen peroxide (H_2_O_2_). (B) superoxide anion (O_2_^–^). Each parameter was measured in triplicate. Statistical differences among groups were assessed using one-way ANOVA followed by Tukey’s multiple-comparisons test. All pairwise comparisons were performed. ns = not significant (p > 0.05), *significant (p < 0.05), **highly significant (p < 0.001). T, tillage method; ST, straw returning; TP, traditional plowing without straw; I, irrigation treatment; NI, normal irrigation, 75% FC; DS, drought stress, 45% FC; V, variety (MC703, A2636, DK159, ZQ1, 303×178, ZJ330).

#### 3.2.2 Straw returning improves H_2_O_2_ and O_2_^-^ under drought stress

**Figure 3:**
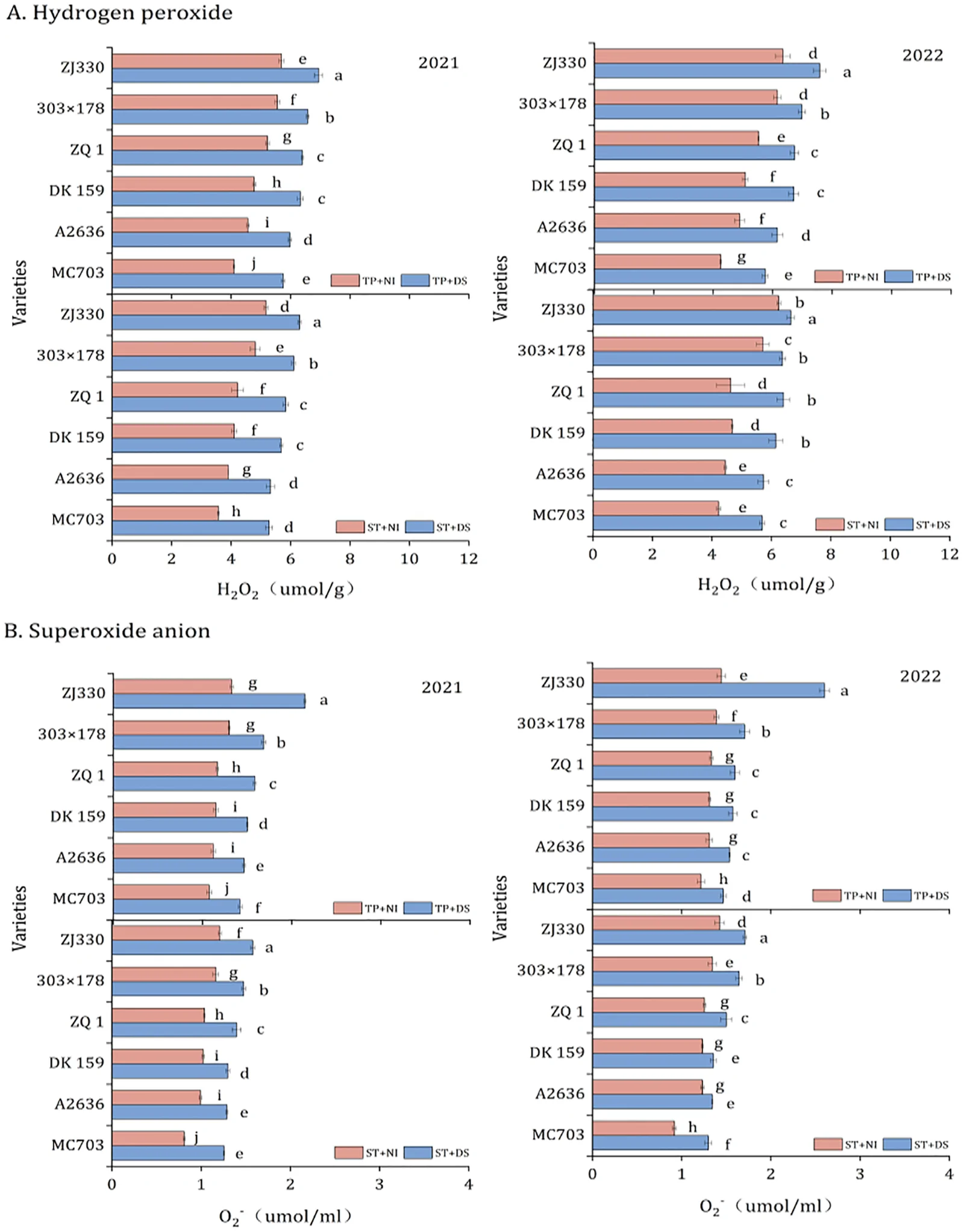
Responses of reactive oxygen species (ROS) to straw returning and irrigation amount. **(A)** hydrogen peroxide (H_2_O_2_). **(B)** superoxide anion (O_2_^-^). For a given treatment, different lowercase letters indicate significant differences among treatments (p < 0.05). Error bars represent standard deviation (SD) based on three replicates. Statistical differences among groups were assessed using one-way ANOVA followed by Tukey’s multiple-comparisons test. All pairwise comparisons were performed. The subplots within each figure were analyzed independently. TP, traditional plowing (without straw); ST, straw returning; NI, normal irrigation (75% FC); DS, drought stress (45% FC).

**Figure 3** illustrates the effects of straw returning (ST) on the accumulation of H_2_O_2_ and O_2_^-^ over the two–year experimental period. Under straw returning (ST) treatment, compared with normal irrigation (75% FC), drought stress (45% FC) increased H_2_O_2_ content by 8.94%–80.56% and O_2_^-^ content by 6.56%–38.44%; Under traditional plowing (TP) conditions, compared with normal irrigation (75% FC), drought stress (45% FC) increased H_2_O_2_ content by 27.45%–61.65% and O_2_^-^ content by 18.35%–47.90%. Under straw returning (ST), drought stress (45% FC) caused a smaller reduction in O_2_^-^ and H_2_O_2_ compared with traditional plowing (TP).

### 3.3 Straw returning enhances the AsA–GSH antioxidant cycle in maize under drought stress

#### 3.3.1 Analysis of variance of non–enzymatic antioxidants in the AsA–GSH cycle

As shown in **Table 5**, the results of the ANOVA for antioxidant compounds and redox ratios associated with the AsA–GSH cycle in maize across the two experimental years indicate that the three main effects–tillage method, irrigation treatment, and variety–all had highly significant effects (P < 0.001) on AsA, DHA, GSH, GSSG, as well as the AsA/DHA and GSH/GSSG ratios. Regarding the two–factor interactions, the effects of tillage × irrigation, tillage × variety, and irrigation × variety were statistically significant only in certain years or for specific AsA–GSH cycle indicators. The three–factor interaction significantly influenced several AsA–GSH cycle indicators. In 2021, it had highly significant effects on AsA and DHA contents and a significant effect on GSH content. In 2022, significant effects were observed on AsA and DHA contents. Moreover, the interaction exerted highly significant effects on GSSG content, the AsA/DHA ratio, and the GSH/GSSG ratio in both experimental years, indicating that the responses of AsA–GSH cycle indicators to the three–factor interaction differed among indicators.

**Table 5:** ANOVA results for non–enzymatic antioxidants across different treatments (F–value).

Year	Source of variation	AsA (ug/g)	DHA (ug/g)	GSH (ug/g)	GSSG (ug/g)	AsA/DHA	GSH/GSSG
2021	Tillage method(T)	2224.02**	911.06**	1508.89**	3518.93**	3344.93**	2277.47**
	Irrigation treatment(I)	22832.30**	4070.12**	5861.95**	15424.35**	15612.51**	9376.24**
	Variety(V)	640.24**	284.80**	362.67**	2610.07**	836.79**	1382.44**
	T×I	67.76**	26.47**	25.09**	24.99**	998.43**	227.46**
	T×V	33.05**	1.67ns	7.14**	24.77**	72.41**	15.55**
	I×V	26.22**	2.89*	3.21*	71.36**	185.57**	167.08**
	T×I×V	12.26**	12.71**	3.64*	49.30**	51.85**	23.58**
2022	Tillage method(T)	570.70**	79.37**	281.41**	1591.64**	322.40**	805.53**
	Irrigation treatment(I)	4527.03**	1035.67**	1530.70**	6420.71**	2466.39**	3494.94**
	Variety(V)	49.98**	114.34**	201.09**	1176.61**	122.90**	693.46**
	T×I	8.22*	4.00ns	0.71ns	8.15*	62.76**	113.53**
	T×V	3.03*	3.98*	9.68**	13.05**	11.49**	8.86**
	I×V	3.28*	4.21*	4.05*	77.98**	20.61**	93.83**
	T×I×V	2.78*	3.62*	1.66ns	20.21**	4.79**	22.10**

(A) ascorbic acid (AsA). (B) dehydroascorbic acid (DHA). (C) reduced glutathione (GSH). (D) oxidized glutathione (GSSG). (E) ascorbic acid/dehydroascorbic acid (AsA/DHA). (F) reduced glutathione/dehydroascorbic acid (GSH/GSSG). Each parameter was measured in triplicate. Statistical differences among groups were assessed using one-way ANOVA followed by Tukey’s multiple-comparisons test. All pairwise comparisons were performed. ns = not significant (p > 0.05), *significant (p < 0.05), **highly significant (p < 0.001). T, tillage method; ST, straw returning; TP, traditional plowing without straw; I, irrigation treatment; NI, normal irrigation, 75% FC; DS, drought stress, 45% FC; V, variety (MC703, A2636, DK159, ZQ1, 303×178, ZJ330).

#### 3.3.2 Straw returning improves non–enzymatic antioxidants in the AsA–GSH cycle under drought stress

**Figure 4:**
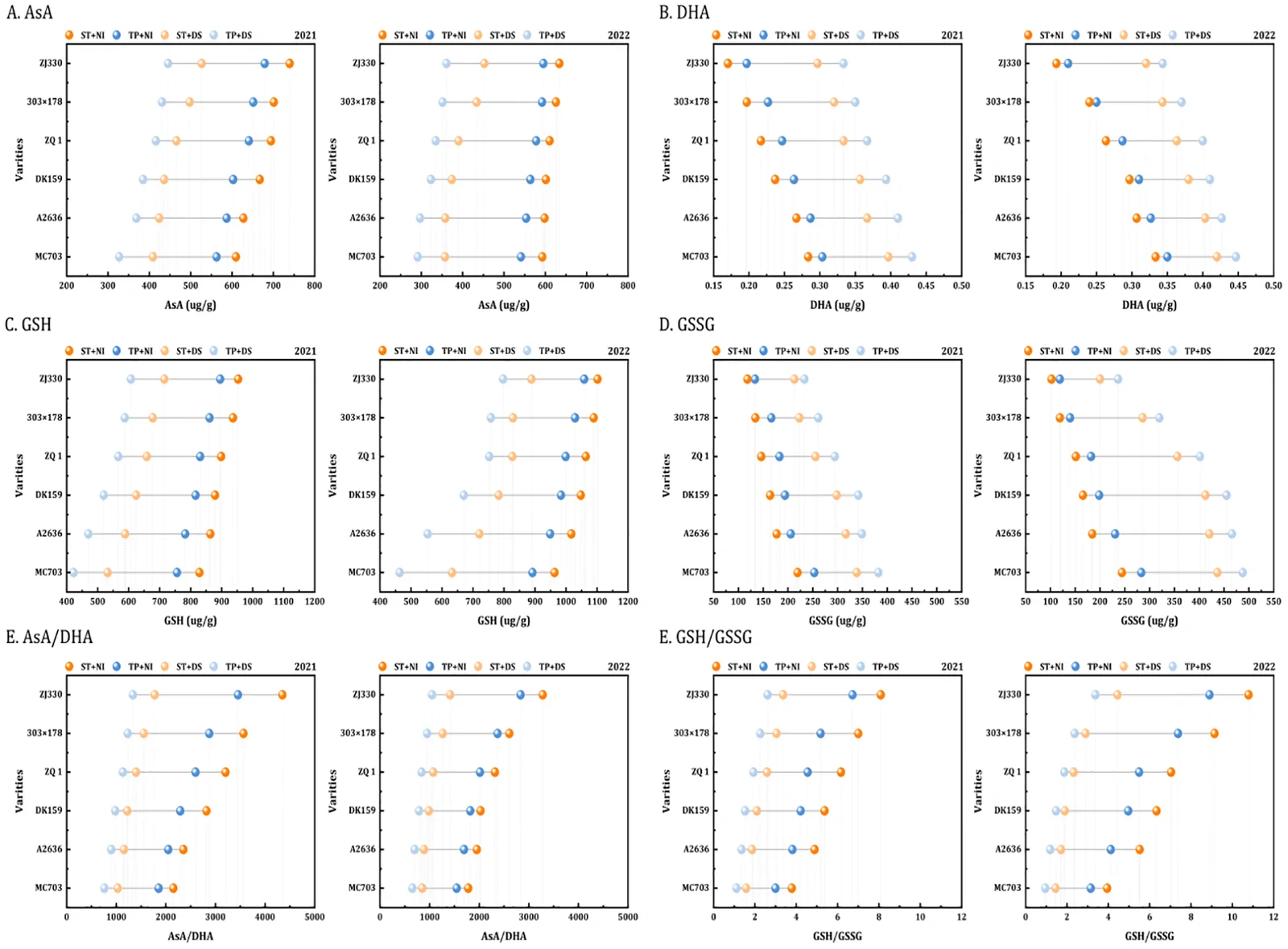
Responses of non–enzymatic antioxidants to straw returning and irrigation amount **(A)** ascorbic acid (AsA). **(B)** dehydroascorbic acid (DHA). **(C)** reduced glutathione (GSH). **(D)** oxidized glutathione (GSSG). **(E)** ascorbic acid/dehydroascorbic acid (AsA/DHA). **(F)** reduced glutathione/dehydroascorbic acid (GSH/GSSG). For a given treatment, different lowercase letters indicate significant differences among treatments (p < 0.05). Error bars represent standard deviation (SD) based on three replicates. Statistical differences among groups were assessed using one-way ANOVA followed by Tukey’s multiple-comparisons test. All pairwise comparisons were performed. The subplots within each figure were analyzed independently. TP, traditional plowing (without straw); ST, straw returning; NI, normal irrigation (75% FC); DS, drought stress (45% FC).

As shown in **Figure 4**, under drought stress (45% FC), straw returning (ST) promoted the accumulation of AsA and GSH, increased the AsA/DHA and GSH/GSSG ratios, and reduced DHA and GSSG contents in both experimental years. Under straw returning (ST) treatment, compared with normal irrigation (75% FC), drought stress (45% FC) decreased AsA and GSH contents by 28.68%–40.21 and 19.35%–35.75%, respectively, and reduced the AsA/DHA and GSH/GSSG ratios by50.89%–59.19% and 56.48%–70.03%, respectively. Meanwhile, DHA and GSSG contents increased by 26.00%–74.51% and 51.49%–149.23%, respectively. Under traditional plowing (TP) treatment, compared with normal irrigation (75% FC), drought stress (45% FC) decreased AsA and GSH contents by 33.91%–46.35% and 24.70%–48.07%, respectively, and reduced the AsA/DHA and GSH/GSSG ratios by56.10%–62.94% and 56.50%–71.16%, respectively. Meanwhile, DHA and GSSG contents increased by 27.62%–39.49% and 51.05%–129.49%, respectively. Changes in ROS levels under drought stress reflect a disruption of cellular redox homeostasis, whereas marked alterations in non–enzymatic antioxidants of the AsA–GSH cycle suggest the activation of antioxidant defense mechanisms in response to stress. Although the overall response of crops to straw returning (ST) under drought stress (45% FC) was relatively limited, significant changes in non-enzymatic antioxidants within the AsA–GSH cycle were still observed, indicating the active regulation of the antioxidant defense system. Furthermore, genetic variation among cultivars contributed to differences in antioxidant capacity, with ZJ330 showing the greatest tolerance to drought stress.

#### 3.3.3 Analysis of variance of antioxidant enzyme activities in the AsA–GSH cycle

As shown in **Table 6**, the results of the ANOVA for antioxidant enzyme activities associated with the AsA–GSH cycle in maize across the two experimental years indicate that the three main effects–cultivation method, irrigation treatment, and variety–all had highly significant effects (P < 0.001) on the activities of APX, MDHAR, DHAR, AAO, and GR. Regarding the two–factor interactions, the effects of tillage × irrigation, tillage × variety, and irrigation × variety were statistically significant in certain years or for specific antioxidant enzyme activities. The three–factor interaction had highly significant effects on the activities of APX, MDHAR, DHAR, and GR in 2021, and significant effects on the activities of MDHAR, AAO, and GR in 2022, indicating that the responses of AsA–GSH cycle related antioxidant enzymes to the three–factor interaction varied among enzyme types and years.

**Table 6:** ANOVA results for enzyme activity across different treatments (F–value).

Year	Source of variation	APX (umol/g)	MDHAR (umol/g)	DHAR (umol/g)	AAO (umol/g)	GR (umol/g)
2021	Tillage method(T)	4000.38**	4169.56**	2260.38**	2130.85**	3109.01**
	Irrigation treatment(I)	18813.00**	20915.17**	7851.41**	6462.88**	12566.04**
	Variety(V)	388.73**	426.03**	553.48**	299.30**	210.97**
	T×I	147.15**	7.27*	3.64ns	0.23ns	70.62**
	T×V	6.73**	5.46**	4.51*	7.34**	22.03**
	I×V	58.80**	5.75**	4.20*	11.61**	72.19**
	T×I×V	14.26**	8.46**	8.74**	1.04ns	7.80**
2022	Tillage method(T)	3199.71**	950.75**	410.77**	4417.84**	1097.69**
	Irrigation treatment(I)	13214.20**	7653.31**	1279.92**	8069.56**	4020.18**
	Variety(V)	1264.73**	100.29**	219.45**	524.61**	28.42**
	T×I	34.37**	39.95**	7.06*	92.32**	0.07ns
	T×V	35.09**	2.49*	2.31ns	4.98**	1.44ns
	I×V	101.17**	5.53**	5.09**	49.68**	258.79**
	T×I×V	18.90**	3.99*	11.43**	2.76*	2.78*

(A) ascorbate peroxidase (APX). (B) monodehydroascorbate reductase (MDHAR). (C) ascorbic acid oxidase (AAO). (D) dehydroascorbate reductase (DHAR). (E) glutathione reductase (GR).Each parameter was measured in triplicate. Statistical differences among groups were assessed using one-way ANOVA followed by Tukey’s multiple-comparisons test. All pairwise comparisons were performed. ns = not significant (p > 0.05), *significant (p < 0.05), **highly significant (p < 0.001). T, tillage method; ST, straw returning; TP, traditional plowing without straw; I, irrigation treatment; NI, normal irrigation, 75% FC; DS, drought stress, 45% FC; V, variety (MC703, A2636, DK159, ZQ1, 303×178, ZJ330).

#### 3.3.4 Straw returning improves enzyme activity in the AsA–GSH cycle under drought stress

**Figure 5:**
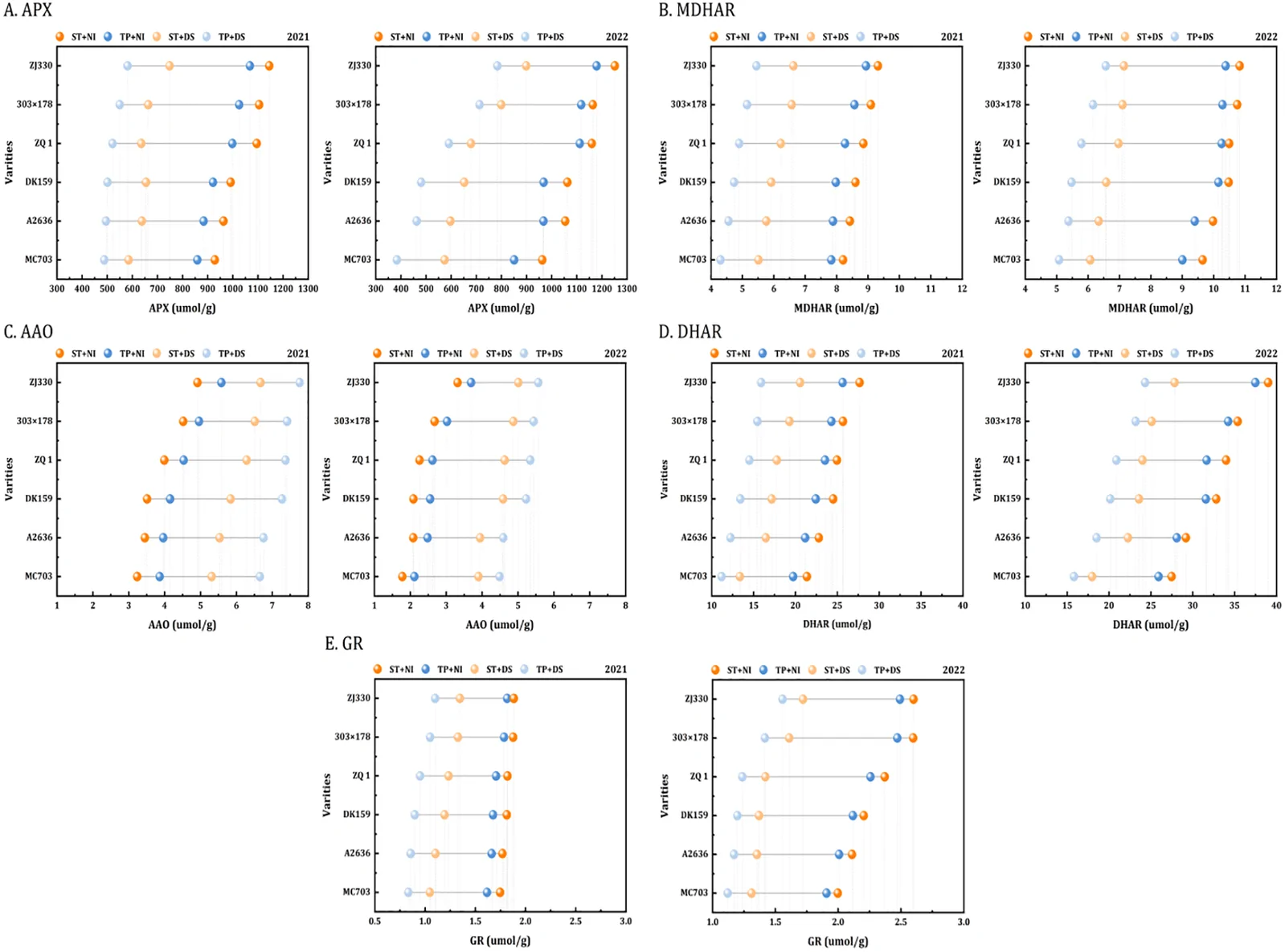
Responses of enzyme activity to straw returning and irrigation amount. **(A)** ascorbate peroxidase (APX). **(B)** monodehydroascorbate reductase (MDHAR). **(C)** ascorbic acid oxidase (AAO). **(D)** dehydroascorbate reductase (DHAR). **(E)** glutathione reductase (GR). For a given treatment, different lowercase letters indicate significant differences among treatments (p < 0.05). Error bars represent standard deviation (SD) based on three replicates. Statistical differences among groups were assessed using one-way ANOVA followed by Tukey’s multiple-comparisons test. All pairwise comparisons were performed. The subplots within each figure were analyzed independently. TP, traditional plowing (without straw); ST, straw returning; NI, normal irrigation (75% FC); DS, drought stress (45% FC).

As shown in **Figure 5**, drought stress (45% FC) significantly decreased the activities of key enzymes involved in the maize AsA–GSH cycle, including APX, MDHAR, DHAR, and GR, while increasing AAO activity in both experimental years. Straw returning (ST) alleviated oxidative stress to some extent, as reflected by increased activities of APX, MDHAR, DHAR, and GR, together with decreased AAO activity. Under straw returning (ST) conditions, compared with normal irrigation (75% FC), drought stress (45% FC) decreased APX, MDHAR, DHAR, and GR activities by 28.18%–43.38%, 27.76%–37.23%, 24.89%–37.50%, and 28.50%–40.08%, respectively, while increasing AAO activity by 35.75%–119.32%. Under traditional plowing (TP) conditions, compared with normal irrigation (75% FC), drought stress (45% FC) decreased APX, MDHAR, DHAR, and GR activities by 33.50%–54.91%, 36.77%–46.08%, 32.26%–46.36%, and37.51%–48.50%, respectively, while increasing AAO activity by 39.07%–112.30%.

On the one hand, straw returning enhanced MDHAR activity, promoted NAD(P)H generation, and accelerated AsA regeneration; On the other hand, straw returning enhanced DHAR activity, enabling the efficient reduction of DHA to AsA using GSH as an electron donor, thereby reducing DHA accumulation and promoting AsA regeneration. At the same time, straw returning enhanced GR activity, facilitating the rapid reduction of GSSG to GSH using NADPH as a reducing power source, thereby maintaining a high intracellular GSH pool and strengthening antioxidant capacity. APX is predominantly localized in chloroplasts, where it functions in close coordination with the AsA–GSH cycle to detoxify H_2_O_2_ generated during photosynthesis. Under different treatment combinations, the magnitude of changes in the activities of AsA–GSH cycle related enzymes varied considerably.

### 3.4 The beneficial role of straw returning in improving yield and yield components of drought–stressed maize

#### 3.4.1 Analysis of variance of yield and yield components

As shown in **Table 7**, the results of the ANOVA for maize grain yield and its yield components across the two experimental years indicate that the three main effects–tillage method, irrigation treatment, and variety–all had highly significant effects (P < 0.001) on ear number (EN), grain number per ear (GNE), hundred–kernel weight (HKW), and grain yield (GY). Regarding the two–factor interactions, tillage × irrigation, tillage × variety, and irrigation × variety significantly affected certain yield traits in some years, with the responses differing among years and traits. The three–factor interaction had highly significant effects on GNE, HKW, and GY in 2021, and significant effects on EN and GY in 2022, indicating that the response to the three–factor interaction varied among yield traits.

**Table 7:** ANOVA results for yield and yield components across different treatments (F–value).

Year	Source of variation	Ear number	Grain number per ear	Hundred–kernel weight	Grain yield
		(1000 fringes/hm^2^)	(grains/fringe)	(g)	(kg/hm^2^)
2021	Tillage method(T)	158.82**	134.32**	159.43**	316.74**
	Irrigation treatment(I)	1185.59**	770.45**	851.83**	979.11**
	Variety(V)	780.97**	198.66**	102.63**	878.52**
	T×I	0.49ns	9.78*	8.17*	30.23**
	T×V	13.07**	4.62*	5.38**	25.87**
	I×V	18.27**	13.83**	26.18**	27.95**
	T×I×V	3.02*	12.21**	10.38**	6.32**
2022	Tillage method(T)	99.66**	9.99*	30.93**	60.02**
	Irrigation treatment(I)	398.75**	45.69**	70.15**	576.89**
	Variety(V)	279.75**	82.02**	1045.30**	1152.24**
	T×I	1.56ns	0.01ns	0.03ns	10.30*
	T×V	4.54*	0.49ns	1.34ns	5.29**
	I×V	17.58**	0.60ns	2.46*	55.09**
	T×I×V	3.09*	0.23ns	0.30ns	3.13*

(A) ear number. (B) grain number per ear. (C) hundred–kernel weight. (D) grain yield. Each parameter was measured in triplicate. Statistical differences among groups were assessed using one-way ANOVA followed by Tukey’s multiple-comparisons test. All pairwise comparisons were performed. ns, not significant (p > 0.05), *significant (p < 0.05), **highly significant (p < 0.001). T, tillage method; ST, straw returning; TP, traditional plowing without straw; I, irrigation treatment NI, normal irrigation, 75% FC; DS, drought stress, 45% FC; V, variety (MC703, A2636, DK159, ZQ1, 303×178, ZJ330).

#### 3.4.2 Straw returning enhances grain yield and yield–related traits in drought–stressed maize

**Figure 6:**
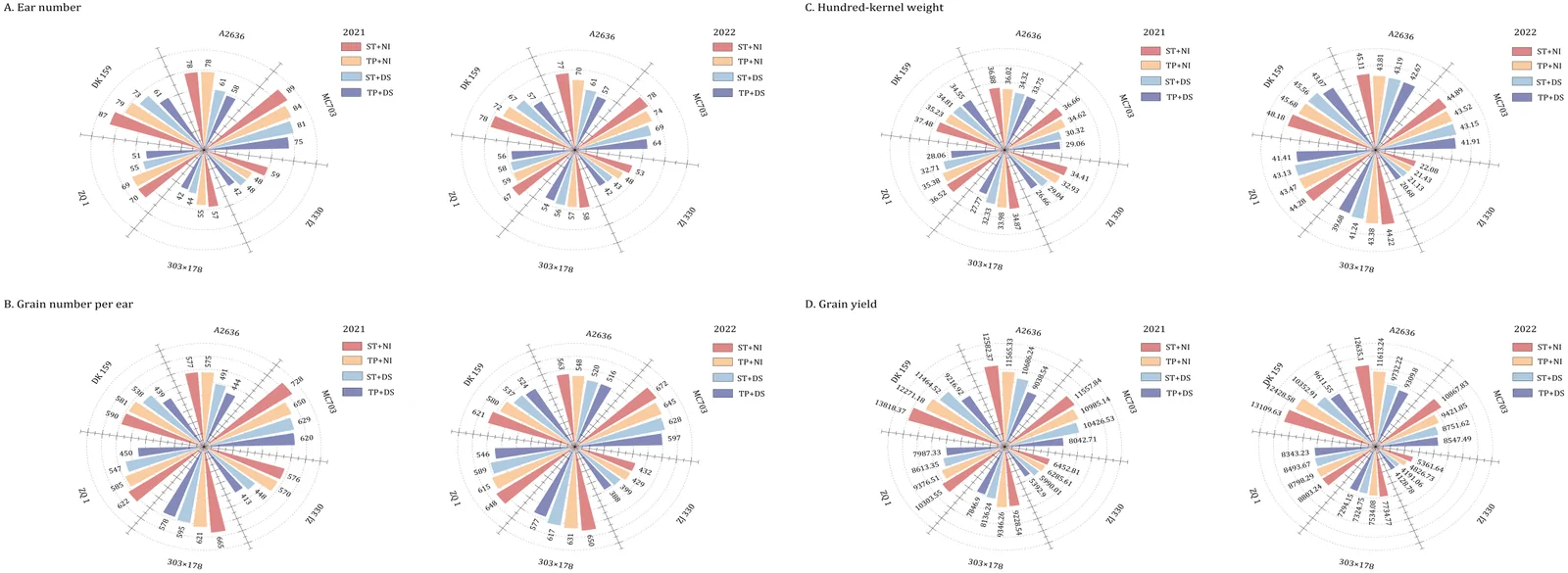
Responses of yield and yield components to straw returning and irrigation amount. **(A)** ear number. **(B)** grain number per ear. **(C)** hundred–kernel weight. **(D)** grain yield. For a given treatment, different lowercase letters indicate significant differences among treatments (p < 0.05). Error bars represent standard deviation (SD) based on three replicates. Statistical differences among groups were assessed using one-way ANOVA followed by Tukey’s multiple-comparisons test. All pairwise comparisons were performed. The subplots within each figure were analyzed independently. TP indicate traditional plowing (without straw), ST indicate straw returning, NI indicate normal irrigation: 75%FC, DS indicate drought stress: 45%FC.

As shown in **Figure 6**, drought stress (45% FC) significantly reduced ear number (EN), grain number per ear (GNE), hundred–kernel weight (HKW), and grain yield (GY) in both experimental years. Straw returning mitigated these adverse effects and improved grain yield and its components across different maize varieties. Under the straw returning (ST) treatment, compared with normal irrigation (75% FC), drought stress (45% FC) resulted in a 2.87%–23.86% reduction in ear number, a 5.10%–22.16% reduction in grain number per ear, a 2.60%–17.29% reduction in hundred–kernel weight, and a 3.52%–22.97% reduction in grain yield. Under traditional plowing (TP), compared with normal irrigation (75% FC), drought stress (45% FC) resulted in a 4.86%–26.06% reduction in ear number, a 4.62%–27.49% reduction in grain number per ear, a 1.93%–20.69% reduction in hundred–kernel weight, and a 3.18%–26.79% reduction in grain yield. Grain yield is a comprehensive indicator of drought tolerance and photosynthetic performance in maize. Under environmental stress, maize activates a range of antioxidant defense mechanisms to alleviate oxidative damage and maintain normal growth and development. Notably, compared with traditional plowing (TP), straw returning (ST) alleviated the reductions in grain yield and its components. This effect can be attributed to improvements in soil physicochemical properties as well as the regulation of the maize antioxidant defense system by straw returning, which together contributed to enhanced yield performance.

#### 3.4.3 Path analysis of maize grain yield and yield component factors

As shown in **Table 8**, the correlation analysis revealed that, in 2021, ear number and hundred–kernel weight were extremely significantly and positively correlated with grain yield (r = 0.828 and 0.731, respectively), whereas grain number per ear was significantly and positively correlated with grain yield (r = 0.423). In 2022, grain yield was extremely significantly and positively correlated with ear number, grain number per ear, and hundred–kernel weight (r = 0.898, 0.575, and 0.841, respectively).

**Table 8:** Path analysis of maize grain yield and yield component factors.

Year	Index	Correlation coefficient	Direct path coefficient	Indirect path coefficient			
				*X1–Y*	*X2–Y*	*X3–Y*	*SUM*
2021	*X1*	0.828	0.649	–	0.352	0.407	0.759
	*X2*	0.423	0.092	0.050	–	0.041	0.091
	*X3*	0.731	0.365	0.229	0.162	–	0.391
2022	*X1*	0.898	0.684	–	0.473	0.518	0.991
	*X2*	0.575	0.588	0.406	–	0.496	0.902
	*X3*	0.841	0.819	0.620	0.690	–	1.310

Y stands for grain yield (GY), X1 stands for ear number (EN), X2 stands for grain number per ear (GNE), X3 stands for hundred–kernel weight (HKW). The “SUM” in the Indirect Pathway Coefficients column represents the total indirect effect of a variable on the dependent variable, obtained by summing its indirect pathway coefficients through all possible intermediate variables.

The path analysis revealed that, in the established path model, ear number, grain number per ear, and hundred–kernel weight all exerted positive direct effects on grain yield, as indicated by their positive direct path coefficients. In 2021, ear number had the largest positive direct effect on grain yield and also exerted positive indirect effects through grain number per ear and hundred–kernel weight, with indirect path coefficients of 0.352 and 0.407, respectively. In contrast, hundred–kernel weight had the second largest positive direct effect on grain yield and exerted positive indirect effects through ear number and grain number per ear, with indirect path coefficients of 0.229 and 0.162, respectively. In 2022, hundred–kernel weight had the largest positive direct effect on grain yield and also exerted positive indirect effects through ear number and grain number per ear, with indirect path coefficients of 0.620 and 0.690, respectively. Ear number had the second largest positive direct effect on grain yield and exerted positive indirect effects through grain number per ear and hundred–kernel weight, with indirect path coefficients of 0.473 and 0.518, respectively.

Based on the results of the two year study, in the constructed path model, both the ear number and the hundred–kernel weight exhibited relatively large positive direct path coefficients, whereas the direct path coefficient of the grain number per ear was comparatively small; In addition, all yield components exhibited varying degrees of positive indirect correlations with one another.

### 3.5 Correlation analysis of soil physical properties with maize antioxidant systems and yield

**Figure 7:**
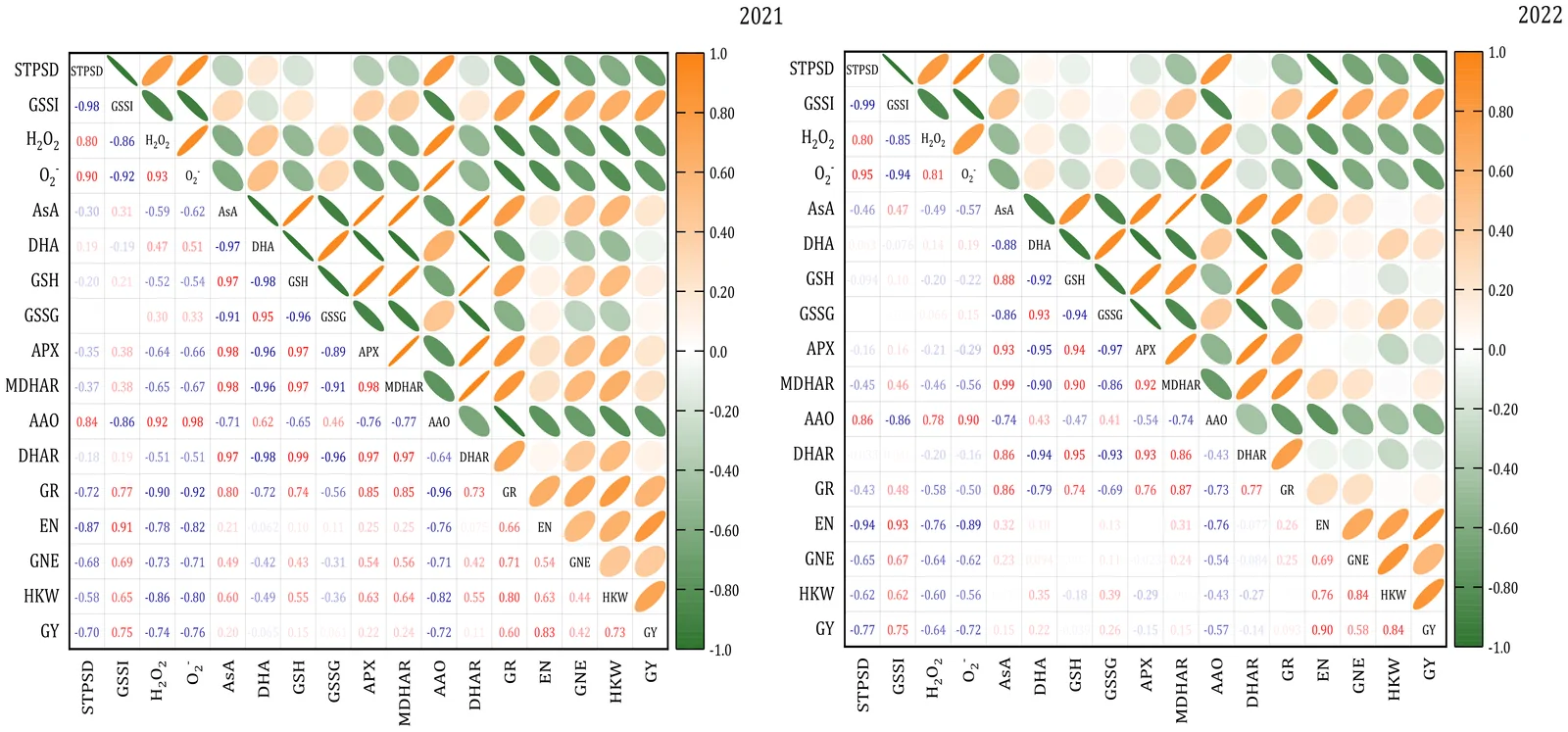
Correlation analysis of soil physical properties with maize antioxidant systems and yield. STPSD indicate soil triple–phase structure distance; GSSI indicate general soil structure index; H_2_O_2_ indicate hydrogen peroxide; O_2_^-^ indicate superoxide anion; AsA indicate ascorbic acid; DHA indicate dehydroascorbic acid; GSH indicate reduced glutathione; GSSG indicate oxidized glutathione; APX indicate ascorbate peroxidase; MDHAR indicate monodehydroascorbate reductase; AAO indicate ascorbic acid oxidase; DHAR indicate dehydroascorbate reductase; GR indicate glutathione reductase; EN indicate ear number; GNE indicate grain number per ear; HKW indicate hundred–kernel weight; GY indicate grain yield.

As shown in **Figure 7**, the soil triple–phase structure distance (STPSD) was positively correlated with ROS (H_2_O_2_ and c) levels in maize leaves, oxidized products (DHA and GSSG) in the AsA–GSH cycle, and AAO activity. Conversely, STPSD was negatively correlated with the GSSI, antioxidant contents (AsA and GSH) in the AsA–GSH cycle, the activities of related enzymes (APX, MDHAR, DHAR, and GR), as well as yield and its components (EN, GNE, and HKW). The above analysis indicates that improved soil physical conditions (i.e., lower STPSD and higher GSSI) are associated with reduced ROS accumulation, lower levels of oxidized products in the AsA–GSH cycle, enhanced antioxidant enzyme activities, and improved yield performance. These results indicate that, following improvements in soil physical properties, the AsA–GSH cycle can maintain redox homeostasis without requiring elevated ROS scavenging capacity, thereby contributing to stable grain yield. However, the results of this study only support a close association between straw returning, which improves soil physical conditions, and the AsA–GSH antioxidant system as well as maize growth performance, while the underlying mechanisms require further investigation and validation.

## 4 Discussion

This study evaluated the effects of different straw returning treatments and irrigation levels on the comprehensive soil physical quality index, maize ROS accumulation, the AsA–GSH antioxidant cycle, and yield, and further elucidated the role of straw application in regulating antioxidant feedback mechanisms in maize under water–limited conditions. The results of this study indicate that, under drought stress (45% FC), soil physical properties were markedly altered, with the three–phase composition progressively deviating from the optimal soil three–phase composition. This was further confirmed by the comprehensive soil quality indices (STPSD and GSSI), where STPSD increased by 1.67%–10.63% and GSSI decreased by 12.43%–37.45%, indicating a significant deterioration in soil structural stability. This study is consistent with previous findings. Xu et al. reported that continuous straw returning for three consecutive years significantly reduced soil bulk density in the 0–20 cm and 20–40 cm layers, promoted soil aggregate formation, and increased soil porosity, thereby improving soil structure. It has been demonstrated in various crops, including wheat, that straw returning improves soil physical properties, thereby promoting crop yield (Xu et al., 2024). Li et al. reported that returning of maize straw into the soil increased the yield of the subsequent wheat crop by 365–844 kg/ha, representing an improvement of 7.1%–11.1% (Li et al., 2025). Zhang et al. reported that straw returning during the growing season increases soil water use efficiency by 15.2%–18.0%, which is accompanied by 13.3%–23.0% increase in yield (Zhang et al., 2017). The findings of this study regarding the positive effects of straw returning on crop yield are consistent with those reported by Li et al. and Zhang et al. Following straw returning (ST), under drought stress (45% FC), the ear number, grain number per ear, hundred–kernel weight, and grain yield of maize increased by 2.63%–19.57%, 1.40%–22.55%, 0.75%–16.54%, and 0.42%–29.64%, respectively. This may be because straw returning increases the input of decomposition products, which promote the formation of stable soil aggregates with soil particles, thereby reducing soil bulk density and increasing porosity; At the same time, the restructuring of the soil pore network further optimized the soil three–phase composition, gradually bringing it closer to the ideal proportions for crop growth and thereby providing a structural basis for high yield formation (Ruch et al., 1989). However, studies on the effects of straw returning on yield components have reported inconsistent conclusions. Turcsanyi et al. found that straw returning did not improve soil fertility or crop yield in dryland soils with low fertility and even showed a potential negative effect (Turcsanyi et al., 2000). Straw returning resulted in an average 1.5% decline in wheat yield over seven years (Wang et al., 2012). These differences may be related to soil fertility, climatic and hydrological conditions, straw decomposition rate, and microbial competition for nutrients. Collectively, these factors regulate the balance between nutrient mineralization and retention during straw decomposition, thereby leading to variations in crop yield. Furthermore, our results indicate that the increase in yield per unit area was primarily attributed to an increase in yield per plant, particularly the ear number and hundred kernel weight. Notably, the DK159 variety exhibited a stronger response to straw returning. As appropriate tillage practices can improve soil physicochemical properties and increase grain yield in intensive maize production systems, it can be concluded that the combination of suitable tillage practices and crop varieties is an effective strategy for maximizing crop yield. Under intensive maize cultivation systems, many studies have suggested that maize straw returning can alleviate constraints imposed by drought stress (Zhang et al., 2017).

In this study, under drought stress (45% FC), the contents of H_2_O_2_ and O_2_^-^ in maize leaves increased by 27.45%–61.65% and 18.35%–47.90%, respectively. This is because, under drought conditions, an imbalance between photosynthesis and respiration leads to over – reduction of the photosynthetic electron transport chain, thereby enhancing electron leakage to O_2_ and generating O_2_^-^. Subsequently, O_2_^-^ is converted into H_2_O_2_ by SOD activity, resulting in excessive accumulation of ROS. The physiological mechanisms underlying ROS accumulation in plants in response to drought stress have been well documented in multiple studies (Peng et al., 2015; He, 2018). Similar enhancement of antioxidant defense systems under drought stress has also been reported in wheat following foliar application of Zn–lysine chelation, where improved antioxidant metabolism contributed to enhanced tolerance against oxidative damage (Ummer et al., 2024). Under the straw returning (ST) treatment, the levels of H_2_O_2_ and O_2_^-^ in maize leaves decreased by 4.09%–34.23% and 1.04%–12 25%, respectively, under drought stress (45% FC). After straw returning, the levels of H_2_O_2_ and O_2_^-^ decreased significantly. This may be attributed to the improvement of soil physicochemical properties and root growth conditions, which alleviated drought–induced stress in maize, mitigated oxidative bursts, and helped maintain membrane integrity.

Biological oxidation is a continuous metabolic process occurring in plants, and the antioxidant system plays a crucial role in maintaining cellular homeostasis and responding to environmental stress (García–Pérez et al., 2025). The plant antioxidant system is broadly divided into enzymatic and non–enzymatic detoxification mechanisms (Xu et al., 2024). The AsA–GSH cycle, a key non–enzymatic pathway for ROS scavenging, operates in close coordination with enzymatic antioxidant systems to eliminate excess H_2_O_2_ generated during photosynthesis, and plays a crucial role in maintaining normal chloroplast function (Cheng et al., 2025). Research by Kaur et al. indicates that the AsA–GSH cycle maintains an optimal redox environment by regulating the AsA/DHA, GSH/GSSG, and NAD(P)H/NAD(P) ratios (Kaur et al., 2023). The key enzymes in this cycle sustain redox homeostasis by facilitating the continuous regeneration of antioxidants, thereby reducing oxidative damage in plants (Wang et al., 2012). Among these, APX is highly sensitive to environmental changes. As the initiating enzyme of this cycle, it scavenges excess H_2_O_2_ by oxidizing AsA; MDHAR and DHAR are two key enzymes in the AsA–GSH cycle responsible for AsA regeneration. They catalyze the reduction of MDHA and DHA back to AsA, respectively; AAO primarily functions in signal transduction processes (Zhang et al., 2017; Cannea and Padiglia, 2025; García–Pérez et al., 2025). Drought stress leads to excessive ROS production. In response, plants enhance AAO activity, promoting AsA oxidation and thereby triggering defense signaling through modulation of the AsA/DHA ratio; As the final enzyme responsible for regenerating GSH and the last step in the cycle, GR activity determines the overall rate of the AsA–GSH cycle (Hasanuzzaman et al., 2019); The implementation of crop straw returning alleviates oxidative damage in crops to some extent by regulating ROS accumulation, as well as modulating the levels of non–enzymatic antioxidants and the activities of enzymes in the AsA–GSH cycle (Sies and Jones, 2020). The results of this study on the AsA–GSH cycle showed that straw returning increased the levels of AsA and GSH in maize leaves under drought stress (45% FC) by 4.76% – 27.99% and 2.70%–16.28%, respectively. The activities of APX, MDHAR, DHAR, and GR increased by 12.09%–49.55%, 8.84%–28.14%, 1.01%–34.51%, and 16.03%–47.82%, respectively, AAO activity decreased by 12.15%–31.36%. Research findings on the effects of straw returning on crop antioxidant systems vary. Research by Li et al. indicates that, under straw returning conditions, antioxidant enzyme activities in cotton decrease, while the level of the oxidative stress marker MDA increases (Li et al., 2009). This may be attributed to the autotoxic effects of phytotoxic compounds released during the decomposition of returned cotton residues (Hu et al., 2023). However, the findings of Fan et al. contradict this view. Their study shows that straw returning enhances soil water–holding capacity and nutrient availability, enabling plants to maintain relatively stable physiological and metabolic functions even under water stress (Fan et al., 2019). In particular, sufficient supplies of N, P, and K provide essential substrates for GSH synthesis, thereby indirectly promoting the proper functioning of the AsA–GSH cycle (Wei et al., 2024). Dai study further elucidates the positive effects of straw returning from the perspective of nutrient supply. The results show that the application of nitrogen fertilizer and organic fertilizer can significantly enhance GR activity (Wang et al., 2025b), which is consistent with the general trend that straw returning improves soil nutrient conditions. This study is consistent with the findings of Fan et al., Dai et al., and others, who reported that straw returning exerts positive effects on plant physiological processes. However, the present study further suggests that straw returning may enhance the efficient functioning of the AsA–GSH cycle under drought stress, thereby maintaining normal maize growth and development, through the combined effects of improved soil physical properties, enhanced antioxidant enzyme activities, and potential regulation by signaling molecules.

## 5 Conclusions

This study investigates the interaction between straw returning and drought stress and their effects on crop yield formation from the perspective of the AsA–GSH cycle, based on improvements in soil conditions. The results indicate that straw returning can effectively improve the soil physical environment under drought stress and is significantly associated with changes in antioxidant compounds and related enzyme activities in the AsA–GSH cycle; it is also closely related to maize growth and yield performance. This suggests that the AsA–GSH cycle may be involved in crop physiological responses to environmental changes and is, to some extent, associated with yield formation. Under the conditions of this study, which included a two–year experiment, a single field site, and a limited number of crop varieties, the ST + 75%FC treatment exhibited superior soil quality, improved redox status, and higher yield levels. In summary, these findings provide a theoretical basis for improving farmland soil quality, enhancing maize drought tolerance, and increasing yield. However, the interactive mechanisms between straw returning and drought stress still require further investigation through multi–site, multi–year, and functional validation experiments to strengthen their generalizability and causal interpretability.

## Data Availability

The original contributions presented in the study are included in the article/supplementary material. Further inquiries can be directed to the corresponding author.
